# The fear of spiders: perceptual features assessed in augmented reality

**DOI:** 10.3389/fnbeh.2024.1355879

**Published:** 2024-02-21

**Authors:** Sergio Frumento, Paolo Frumento, Marco Laurino, Danilo Menicucci, Angelo Gemignani

**Affiliations:** ^1^Department of Surgical, Medical, Molecular and Critical Area Pathology, University of Pisa, Pisa, Italy; ^2^Pisa Research Area, National Research Council (CNR), Pisa, Italy

**Keywords:** arachnophobia, spider phobia, texture, movement pattern, body proportions, perception

## Abstract

**Background:**

Persons with specific phobias typically generalize the dangerousness of the phobic animal to all members of its species, possibly as a result of malfunctioning brain circuitry normally providing quick and dirty identification of evolutionary-relevant stimuli. An objective assessment of which perceptual features make an animal more or less scary to phobic and non-phobic people would help overcome the limitations of the few studies available so far, based on self-reports.

**Objective:**

To achieve this aim, we built an augmented reality setting where volunteers with different levels of fear of spiders were asked to make holographic spiders that look either dangerous or harmless. To reach this goal, a computerized interface allowed participants to modify the spider’s perceptual features (hairiness, body/leg size, and locomotion) in real time.

**Results:**

On average, the dangerous spiders were made hairy, thick, and moving according to spider-like locomotion; coherently, the harmless spiders were made hairless, slim, and moving according to a butterfly-like locomotion. However, these averaged preferences could not fully describe the complex relationship between perceptual preferences with each other and with arachnophobia symptoms. An example of a key finding revealed by cluster analysis is the similarity in perceptual preferences among participants with little or no fear of spiders, whereas participants with more arachnophobia symptoms expressed more varying preferences.

**Conclusion:**

Perceptual preferences toward the spider’s features were behaviorally assessed through an observational study, objectively confirming a generalization effect characterizing spider-fearful participants. These results advance our knowledge of phobic preferences and could be used to improve the acceptability of exposure therapies.

## Introduction

“All spiders are equally scary, but some spiders are more equal than others.” This paraphrase from Orwell’s Animal Farm may represent the summary of a few studies that investigated the features of spiders that make them fearful (Davey, 1991; Lindner et al., 2019). Although any spider can somehow elicit a phobic reaction in spider-fearful people, participants in some studies have referred to being specifically more sensitive to some perceptual features (in particular, the locomotion pattern; Davey, 1991; Lindner et al., 2019) than to others. These self-reported preferences found indirect confirmation in behavioral studies describing attentional or interpretational biases for spiders depending on their movement trajectory, either actual (Basanovic et al., 2017) or perceived (Riskind et al., 1995).

The features that make spiders fearful and/or disgusting have been proposed to be cross-cultural characteristics (Davey et al., 1998) that distinguish them from similar animals (Landová et al., 2021), including from those sharing comparable dimensions and poisonousness (e.g., bees and wasps, in Gerdes et al., 2009); however, Frynta et al. (2021) suggested that arachnophobia could derive from a more evolutionarily-relevant fear for scorpions.

Interestingly, these preferences do not seem to have any rational ground (e.g., they are not specific characteristics of poisonous spiders), coherently with the description of phobia adopted in DSM 5: a disorder characterized by an exaggerated fear of a certain stimulus or animal (American Psychiatric Association, 2013). Despite the lack of rational links between perceptual features of spiders and their dangerousness, arachnophobia is ranked as one of the most common specific phobias (Eaton et al., 2018). The higher prevalence of phobias related to evolutionary-relevant stimuli (e.g., spiders and snakes) than to culturally relevant stimuli (e.g., atomic bombs and guns) supports the idea of an innate preparedness for phobias (Seligman, 1971).

The idea that specific phobias are hardwired in the nervous system (LeDoux, 1994) fits with the evidence that brain circuits related to the categorization of spiders differ from those related to the categorization of snakes (Taschereau-Dumouchel et al., 2018). The existence of a brain circuitry decoding for specific animals implies that the perceptual features mostly characterizing evolutionary-relevant stimuli could trigger a stronger fear response than generic features shared with evolutionary-irrelevant stimuli. This could happen at an unconscious level, with relevant clinical implications for the optimization of exposure therapies (Taschereau-Dumouchel et al., 2018; Baroni et al., 2021; Frumento et al., 2021, 2022; Cesari et al., 2023). When facing a spider, emotions and the consequent behaviors could be elicited with little or no role of cognitive, conscious processes. Deepening the role of consciousness in coupling emotions and behaviors could help understand specific phobias and improve therapeutic approaches.

In line with this scope, it has been recently proposed that investigating the perceptual features that make spiders scary (Frynta et al., 2021) and checking for their possible association with phobic symptoms (Lindner et al., 2019) could shed light on the evolutionary origins of spider phobia. This association appears to be non-linear, suggesting a ceiling effect (Lindner et al., 2019); most participants rated the movement pattern as the perceptual feature that is most responsible for the perceived dangerousness of spiders (Lindner et al., 2019). However, the authors recognize that these results were limited to self-reports related to imaginary spiders, for which only one feature (i.e., locomotion) was investigated.

To overcome the limitations affecting all studies that investigated differences in scariness due to perceptual features of phobic stimuli, an ecological experimental setting was developed in augmented reality, allowing participants to manipulate various features of hyper-realistic virtual spiders in order to make them look dangerous or harmless. Primarily, volunteers were characterized by different levels of fear of spiders, and their preferences were analyzed taking into account the lack of linear association with phobic symptoms previously reported (Lindner et al., 2019).

## Methods

### Study design

The present study can be intended as an observational, cross-sectional study. We did not provide a direct experimental manipulation, even if the experimental setting represents a situation somewhat different from the typical examples presented when describing observational studies.

Indeed, the present study assessed the behavioral preferences expressed by more or less spider-fearful participants toward perceptual features of virtual spiders without repeating the assessment. Given these bases, we followed the STROBE recommendations for a standardized description of observational studies (von Elm et al., 2008).

### Participants

The study was approved by the local Ethical Committee with protocol 0025068/2019.

A total of 56 participants (41 women; mean age = 26.4 years, SD = 5.2) were selected among persons who replied to Internet ads searching for volunteers in a psychological experiment about the fear of spiders. The imbalance in favor of female participants (representing 28.1 and 71.9% of the sample, respectively) is in line with the higher prevalence of this specific phobia among women (Eaton et al., 2018). After expressing the intention to participate and signing the informed consent, volunteers were requested to fulfill two preliminary questionnaires in order to be admitted to the experimental session: the Spider Phobia Questionnaire (SPQ; Klorman et al., 1974) to quantify their level of spider fear and the Symptom Checklist 90-R (SCL-90-R; Derogatis, 1994) to evaluate the presence of exclusion criteria (i.e., psychopathological symptoms that could possibly affect the results; the subscale of phobic anxiety was ignored to not exclude phobic participants from the study). The choice of the SPQ was based on the scientific literature focused on spider-fearful and arachnophobia participants (e.g., Frumento et al., 2021); it was preferred over a similar test (the Fear of Spiders Questionnaire; see Muris and Merckelbach, 1996 for a comparison) whose items refer to a time period more recent than SPQ items, whereas our interest concerned long-term spider fear. The choice of the Symptom Checklist 90-R was based on its potential to reveal psychopathological symptoms in clinical as well as non-clinical or sub-clinical populations (Derogatis, 1994). Finally, only subjects with normal or corrected-to-normal vision were included in the analyses.

This final sample size guarantees that if the true correlation between two variables is 0.4, the null hypothesis of non-correlation will be rejected with a power of 0.8 at a significance level of 0.05.

### Experimental setting

**Figure 1:**
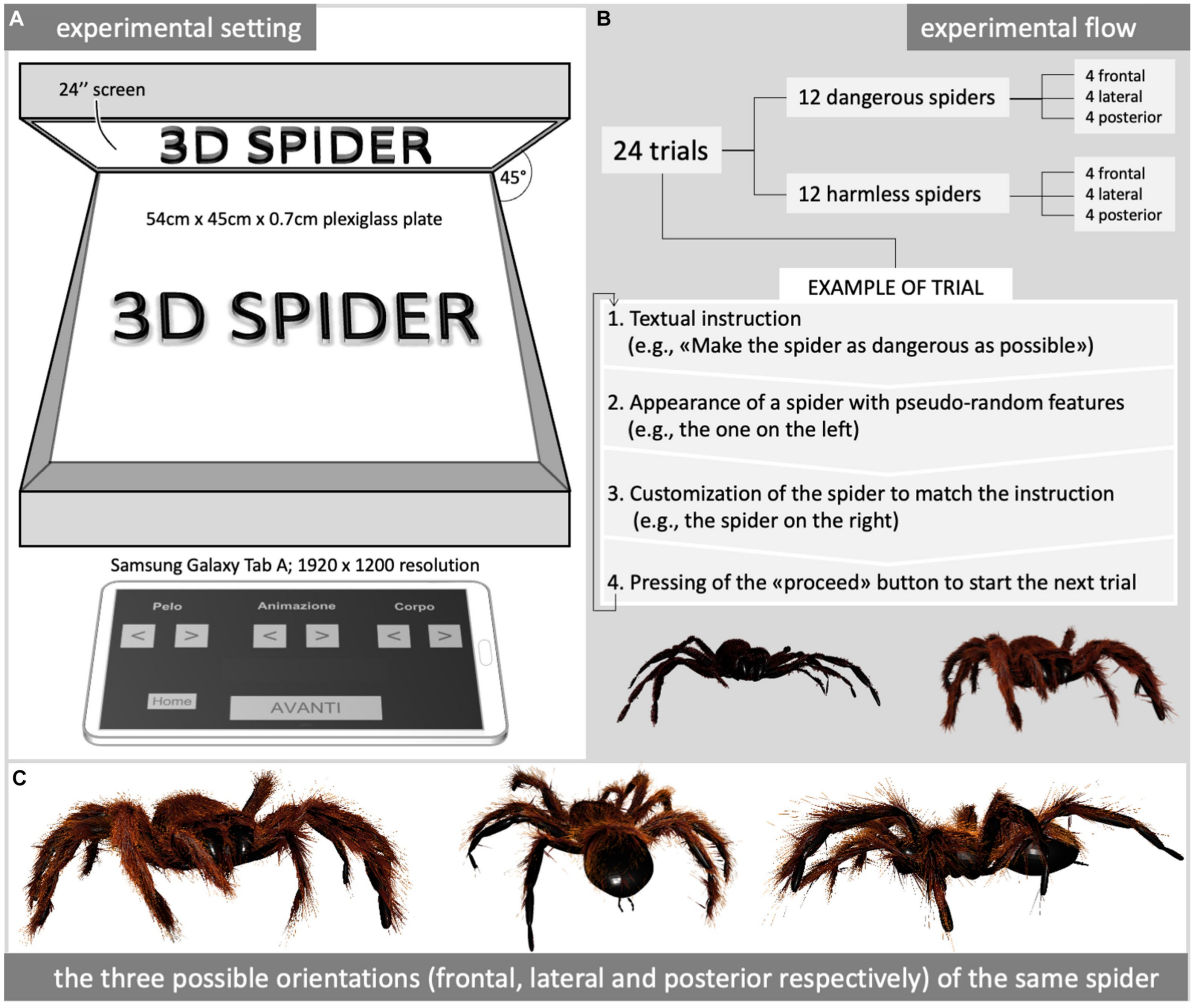
Experimental setting **(A)**, procedure **(B)**, and some examples of stimuli **(C)**. The tablet’s interface **(A)** shows the Italian labels translatable as “hair,” “locomotion,” and “body/leg” above a couple of arrows used by the participant to customize the corresponding perceptual feature accordingly with each trial’s instruction **(B)**. All the exemplification spiders shown in **(C)** were crawling according to a spider-like locomotion pattern.

The experiment was conducted in an augmented reality environment (Figure 1A) consisting of (i) a holographic screen showing the virtual tridimensional spiders and (ii) a tablet showing the interface used by participants to modify the perceptual features of such spiders in real time. In detail, the holographic screen appeared as a 51.5 × 60.5 × 38 cm black box with a 54 × 45 cm plexiglass plate arranged at 45° with respect to the participant’s point of view. Hidden from the direct view of volunteers, this black box concealed a 24″ monitor (Asus VG248; 1920 × 1,080 resolution) oriented face down; its content was perceived as tridimensional thanks to a refraction effect made possible by the physical properties of the plexiglass plate. Being based on a holographic illusion, the whole augmented reality environment was located in a completely dark room to prevent unpredictable differences in luminosity from affecting the perception of tridimensional spiders (Figure 1A).

The final visual effect consisted of a spider crawling in the middle of the dark room where the experiment was carried out.

### Stimulus material

Virtual spiders were created, shown, and modified by participants in real time by means of dedicated software developed by the Cyberfreak Creative Studio,^1^ based on the Unity engine (running in Windows 10 Home 64 bit on an Intel i3 PC mounting 8 GB RAM and an Intel(R) HD Graphics 4,000 video card). The software allowed volunteers to customize spiders with a simple interface provided by a touchscreen 10.1″ tablet (Samsung Galaxy Tab A; 1920 × 1,200 resolution - Figure 1A).

The participants were able to independently modify three perceptual features (the hairiness, the leg movement pattern, and the body/leg proportions) of the virtual spider to make it as dangerous or as harmless as possible, coherently with the instruction given before each trial (Figure 1B). Each perceptual feature could be set to one of 10 possible degrees corresponding to two extremes and their intermediate steps: the hairiness could be set to make the spider completely furry, completely hairless, or eight intermediate steps between these two extremes; the leg movement pattern could be fully spider-like, fully butterfly-like (flying), or eight intermediate steps between these two extremes; the body/leg proportions could be set to make the spider thick with short stubby legs, slim with long skinny legs, or eight intermediate steps between these two extremes.

Once a button was pressed, the virtual spider’s appearance was consequently changed in real time. The starting value of each feature was randomized, that is, each trial showed a different spider at the beginning.

### Procedure

The volunteers were welcomed in the experimental room, where they could sit in a comfortable armchair in front of the holographic screen. The position of this customizable chair—suited to a transcranial magnetic stimulation device and similar to the kind of chair that can be found at every dental practice—was adapted to each participant’s needs to allow a standardized view of a holographic stimulus different from those used in the experiment (a deer): before each experiment, the experimenter was able to adjust (1) the height of the sitting participant, (2) the inclination of the backrest, and (3) the position of the headrest.

Paraphrasing the description reported in the informed consent, the experimenter explained that the volunteer was asked to modify some perceptual features (the hairiness, the body/leg proportions, and the locomotion pattern) of 24 virtual spiders according to the instructions preceding each trial, asking to make the spider either as dangerous or as harmless as possible (Figure 1B). Of these 24 virtual spiders, eight were approaching the participant, eight were running away from the participant, and eight were sliding from right to left based on the participant’s perspective (Figure 1C).

The experimenter showed the tablet interface to modify the virtual spiders, explaining its functioning. As soon as the customization of the virtual spider fitted the instructions given, the participant had to press a button for saving these preferences and then showing the instructions for the following trial. Once assured that the procedure had been correctly understood, the experimenter left the room and turned off the light, instructing the participant to call him back at the end of the experiment.

### Data analysis and representation

Analysis was conducted using R (R Core Team, 2021). Data and scripts are publicly available at the Open Science Framework repository;^2^ the Unity software used in the experiment is publicly available on the GitHub repository.^3^

For each trial, three values describing the participant’s choices about the hairiness, the movement pattern, and the body/leg ratio were recorded, along with a further value indicating the spatial orientation of the spider shown in the trial. Since the mere descriptive statistics (e.g., grand average of the mean value of the hairiness for dangerous spiders) could fail to describe each participant’s preferences and their relationship with spider fear, the raw data were pre-processed, analyzed, and represented according to the following steps:

- In order to obtain robust estimates of preferences for each participant, a specific function computing the mean of the modal part of the distribution (from now on, MMPD function) was implemented. This function is meant to synthetize each preference in a parameter that is more representative of the participant’s actual choices, minimizing the effect of observations that accidentally deviated from the bulk of distribution due to confounding factors (e.g., mistakes and boredom after the multiple trials that composed the experiment). For each participant and instruction (i.e., «Make the spider most dangerous/most harmless as possible»), this function first identifies whether the feature values of the trials (ranging from 0 to 1) are more frequently above or below 0.5 (the values exactly corresponding to 0.5 were ignored in this step). Only the values falling in the most frequent range were averaged, including the values exactly equal to 0.5. While this procedure allows the extrapolation of a value representative of the most consistent preference expressed by each subject, it could also increase the clusterization effect by polarizing preferences compared to those that are more continuous when summarized through the mean or the median: for this reason, the results obtained using this function were compared with those obtained using the mean and the median values calculated over the trials to represent each participant’s preferences.
- To estimate individual incoherency in the preferences expressed over the trials, for each perceptual feature, the absolute value resulting from the difference between the average value related to dangerous spiders and the average value related to harmless spiders was calculated. In order to obtain an index ranging from 0 to 1 (corresponding to minimum and maximum incoherency, respectively), the formula used was x = |p^D^ + p^H^ – 1|, where p^D^ and p^H^ represent the preference expressed for dangerous and harmless spiders, respectively. A perfectly coherent participant would give harmless spiders an appearance specular to that given to dangerous ones (and vice versa), thus resulting in an incoherency index equal to 0. On the other hand, an incoherent participant could rate a similar feature to characterize both harmless and dangerous spiders, thus resulting in a difference distant from 0;
- An association analysis was performed to measure the possible relationships between each couple of variables (SPQ score, dangerous perceptual features, and harmless perceptual features). The outcomes of this analysis were reported in two complementary figures for the sake of completeness and clarity:
- a correlation matrix highlighting the intensity and direction of each possible correlation between a couple of variables. Each piece of information is reported both graphically (as a circle the color and dimension of which convey the intensity and direction of the correlation) and as a *value of p* related to Spearman’s correlation coefficient;
- a plot showing the distributions of preferences expressed for both instructions by each participant and perceptual feature (vertical axis) as a function of the level of fear expressed by the SPQ score (horizontal axis). Associations with the SPQ scores were tested with a regression model in which the effect of SPQ on the conditional mean of each response was allowed to deviate from linearity; to this goal, the regression equation included the basis of a natural cubic spline with one internal knot at the empirical median of SPQ (Schoenberg, 1988). In addition, also the log-variance was modeled as a non-linear function of SPQ, using the same spline-based approach and assuming a normal distribution. Estimation was carried out by maximum likelihood. Finally, a hierarchical cluster analysis (dendrogram) with distance calculated using the centroid method applied to the standardized responses was carried out for each harmless or dangerous perceptual feature to add a clusterization to the visual representation of participants’ preferences. We chose among 2, 3, or 4 clusters by minimizing the Bayesian Information Criterion (BIC) of a multivariate normal distribution. The cluster analysis should be intended to serve as a useful descriptive tool that may improve a correct—albeit inevitably subjective—interpretation of the results. This implies caution in drawing far-fetched conclusions.

## Results

Among the 56 volunteers initially recruited, 10 were excluded based on the clinical threshold of psychopathological symptoms different from specific phobias revealed by the SCL-90-R. One participant was further excluded for suspending the ongoing session due to an unsustainable aversiveness for the experimental setup. The final sample considered for analyses was then reduced to 45 participants (12 men, 33 women; mean age = 26.2 years, SD = 5.5), with a level of arachnophobia symptoms—as measured through the SPQ—continuously spread from 0/30 to 26/30. The higher rate of females (73.3% of the final sample) is consistent with the higher prevalence of specific phobias in women (Eaton et al., 2018).

Table 1 summarizes the perceptual preferences expressed by all participants about dangerous and harmless spiders, comparing the three statistics (mean, median, and MMPD function) and the incoherency value described in section 2.5. Spiders modified to be as fearful as possible (red-filled cells in Table 1) were hairy, thick, and moving in a spider-like pattern. Particularly, the average preferences expressed by participants to make spiders as harmless as possible (green-filled cells in Table 1) resulted in spiders that were hairless, slim, and moving in a butterfly-like pattern. Incoherency between the preferences expressed for harmless and dangerous spiders was low for all features.

**Table 1:** Grand averages (standard deviations in the brackets) of perceptual preferences among all participants expressed through different statistics.

	Hairiness (0 = hairy; 1 = hairless)		Body/leg size (0 = thick; 1 = slim)		Locomotion (0 = butterfly-like; 1 = spider-like)	
	Dangerous	Harmless	Dangerous	Harmless	Dangerous	Harmless
Mean	0.35 (0.22)	0.74 (0.22)	0.37 (0.23)	0.69 (0.24)	0.75 (0.19)	0.4 (0.26)
Median	0.32 (0.28)	0.76 (0.27)	0.32 (0.29)	0.71 (0.29)	0.77 (0.23)	0.32 (0.26)
MMPD function	0.33 (0.28)	0.75 (0.28)	0.34 (0.31)	0.71 (0.29)	0.77 (0.21)	0.4 (0.32)
Preference incoherency	0.12 (0.11)		0.12 (0.11)		0.17 (0.17)	

Each perceptual feature could be customized to reach a minimum arbitrary unit of 0 or a maximum arbitrary unit of 1 alongside 8 further possible steps, 0 and 1 representing maximum and minimum hairiness; butterfly-like and spider-like movement patterns; thick body/leg proportions and slim body/leg proportions, respectively. The function computing the mean of the modal part of distribution (MMPD) is detailed in the methods. The last row (Preference incoherency) reports the absolute value resulting from the difference between each dangerous and each harmless perceptual preference: the closer to 1, the higher the incoherency between preferences.

**Figure 2:**
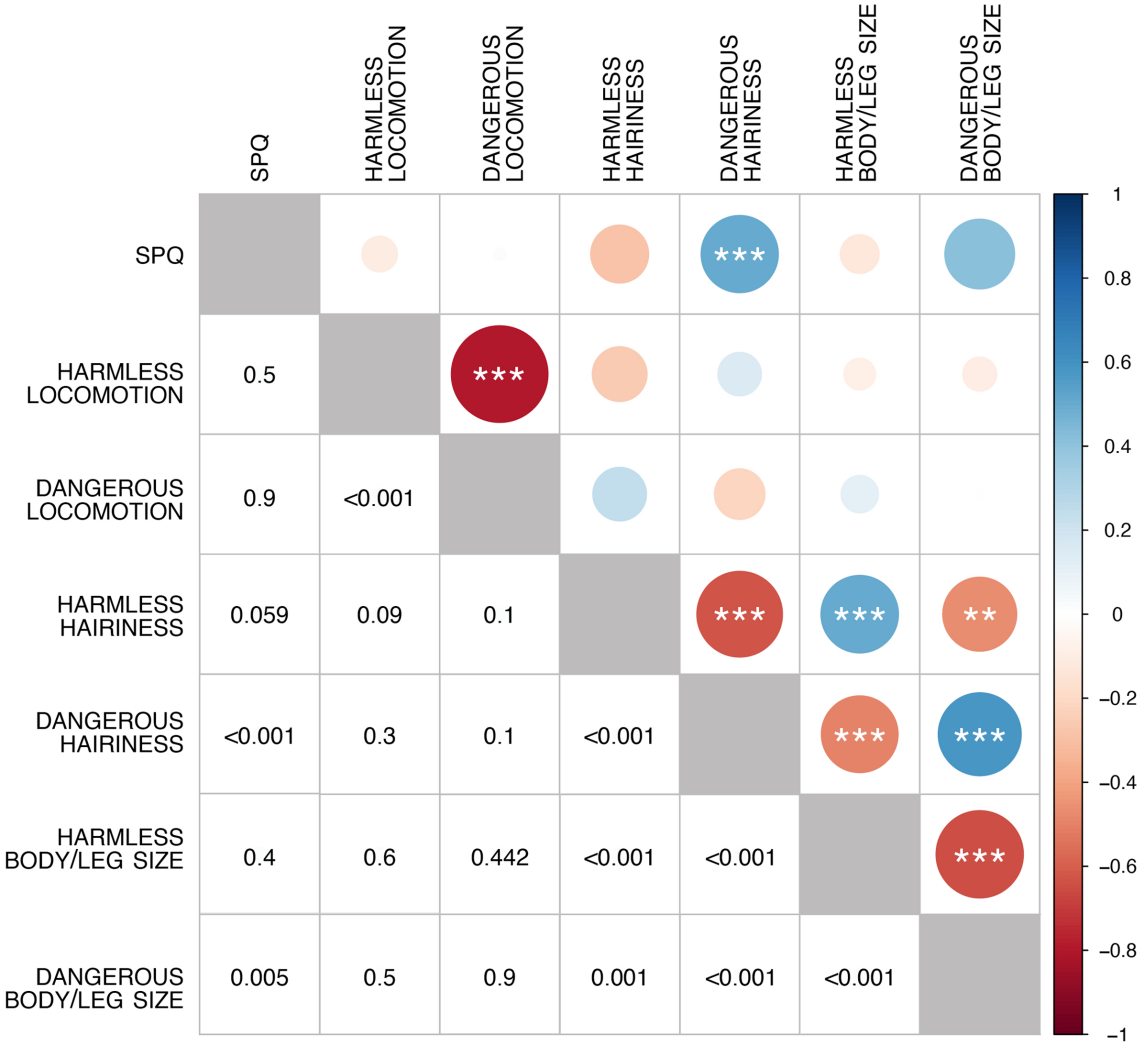
Correlation matrix for all factors (perceptual features, dangerousness, and fear of spiders). The color of the circles represents the sign of the correlation according to the legend on the right. The bottom-left half of the matrix reports the *p*-values represented in the top-right half. Diagonal circles represent the perfect correlation between each variable and itself, giving a visual parameter of the maximum diameter possible.

The correlation matrix of the three features (the hairiness, the body/leg size, and the locomotion pattern, each in both its dangerous and harmless variations) and the fear of spiders revealed the existence of significant relations (Figure 2). In particular, the preferences expressed for each feature when making it dangerous or harmless resulted in being significantly and negatively correlated; moreover, a positive correlation between the level of fear of spiders (SPQ) reached (*p* < 0.001) and approached (*p* = 0.05) significance with the preferences expressed about the hairiness and the body/leg size of dangerous spiders; the preferences expressed for the hairiness and body/leg size of both the dangerous and the harmless spiders were significantly correlated (see Figure 2).

**Figure 3:**
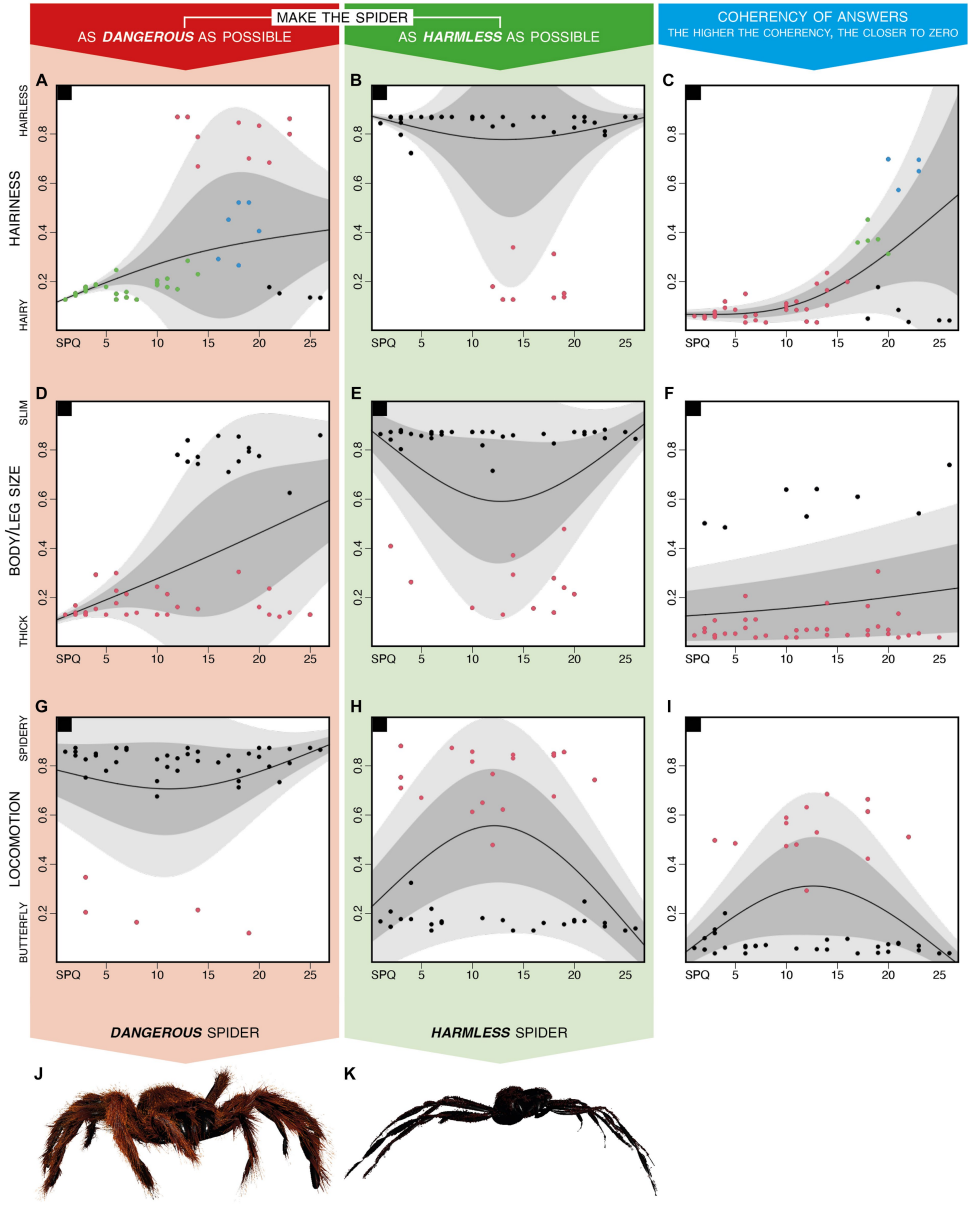
Distributions of perceptual preferences modeled with the normal heteroskedastic spline-based model. Distributions of preferences expressed by each participant to the 12 trials asking to make the spider as fearful as possible (red background column) and to the 12 trials asking to make the spider as harmless as possible (green background column), as summarized through the MMPD function described in methods. For each plot, a solid black line describes the mean for each point; a dark-gray area describes the 1st to the 3rd quartile; a light-gray area indicates the 1st to the 9th decile. The plots on the 1st row **(A–C)** are related to the spider’s hairiness; those on the 2nd row **(D–F)** are related to the spider’s body/leg ratio; those on the 3rd row **(G–I)** are related to the spider’s movement pattern. The pictures on the 4th row are a graphical representation of the perceptual features averaged for all participants realized by inputting in the presentation software the values reported in the third row of Table 1. Note that the movement pattern is spider-like for dangerous spiders **(J)** and butterfly-like for harmless spiders **(K)**.

Figure 3 shows individual data (each dot corresponds to a subject), previously summarized in Table 1, and models them with a normal heteroskedastic spline-based model to (1) deepen the relations between each perceptual preference and the level of fear of spiders (SPQ score) and (2) further integrate this information with the results of a hierarchical cluster analysis. In fact, for each row of the figure, two plots describe how each feature was modified to create dangerous (Figures 3A,D,G) or harmless (Figures 3B,E,H) spiders. A third plot describes the distribution of inter-individual incoherency in perceptual preferences in relation to the level of spider fear (Figures 3C,F,I). Finally, each plot indicates with different colors of the dots, the presence of multiple groups resulting from cluster analysis.

Integrating the information conveyed by Figures 2, 3 shows that the preferences expressed about the hairiness and the body/leg size of dangerous spiders are very similar among participants with low SPQ scoring, compared to those with high SPQ scoring (Figures 3A,D). The cluster analysis based on each feature (i.e., the hairiness, the body/leg size, and the locomotion pattern) and each instruction (i.e., making the spider as dangerous or as harmless as possible) revealed that in most cases (Figures 3B,D,E,G,H), two distinct groups were characterized by preferences polarized on the two possible extremes. Finally, the inter-individual incoherency between hairiness preferences related to dangerous and harmless spiders showed that the participants with higher levels of fear of spiders were less coherent than those with low or null levels of fear of spiders (Figure 3C). On the other hand, the incoherency between preferences concerning the body/leg size and locomotion was not affected by the fear of spiders (Figures 3F,I).

It is worth noting that Figure 3 represents the preferences calculated through the MMPD function described in the “Data analysis and representation” section; however, preferences summarized through means (Supplementary Figure S3) or medians (Supplementary Figure S4) show a comparable distribution. Figure 3 uses different colors to represent a clusterization based on one factor per plot, whereas the results related to other clustering criteria are reported in Supplementary materials. Supplementary Figures S5–S7 show the same data of Figure 3 clustered for all factors (the three perceptual features and the SPQ score), all perceptual features, and the harmless/dangerous version of each feature, respectively.

Finally, the orientation of spiders (frontal, lateral, or posterior) resulted in slightly different preferences about the hairiness of dangerous spiders, which was on average lower for spiders approaching the participant than for those sliding or withdrawing: preferences about the body/leg size and the locomotion were almost overlapped regardless of orientation (Supplementary Figures S1, S2).

## Discussion

The present study reports behavioral evidence that specific preferences in perceptual features can make a virtual spider look more or less dangerous/harmless. Importantly, some of these preferences are correlated with the level of fear of spiders.

On average (Table 1), fearful spiders were hairy, thick, and moving according to a spidery pattern (Figure 3L); particularly, harmless spiders were hairless, slim, and moving according to a butterfly-like pattern (Figure 3M). However, the relationship between phobic symptoms and sensitivity to each perceptual feature is more nuanced. In particular, Figure 2 reports significant correlations between many couples of variables, the most interesting of which showed that (1) less spider-fearful participants found hairy spiders to be more dangerous, and (2) spiders modified to look dangerous were both thick and hairy, despite most of the other features are mutually independent.

In fact, the unrecognizable grouping resulting from the different clustering criteria shown in Supplementary Figure S7 suggests that the preferences shown for each feature are mutually independent (i.e., dangerous spiders can be slim or thick regardless of their hairiness). To the contrary, Figures 2, 3C,F,I (in particular, significant correlations between each feature’s harmless and dangerous preferences) show that each perceptual feature was characterized by a high internal coherency (i.e., participants who made harmless spiders slim also made fearful ones thick): hairiness’ coherency lowered at the increase of fear of spiders (Figure 3C), thus exemplifying the generalization effect that characterizes phobic patients according to DSM 5 (American Psychiatric Association, 2013).

When clustering criteria were limited to each specific feature (e.g., the hairiness of dangerous spiders), two groups of participants could be recognized as expressing preferences polarized on the two possible extremes (e.g., Figure 3B), corroborating the lack of a linear relationship between arachnophobia symptoms and features’ preferences previously reported by Lindner et al. (2019). The group membership was in most cases not related to the level of fear of spiders; however, for both the body/leg size of dangerous spiders (Figure 3D) and the hairiness of harmless spiders (Figure 3B), the minority group was entirely composed of the participants with mild or severe (SPQ score ranging between 10 and 30) phobic symptoms.

In contrast with this polarization of preferences, the hairiness was the feature revealing the smallest differences among clusters (Figure 3A) and the higher incoherency in answers (Figure 3C): these results—and, to a lesser extent, those related to the body/leg size (Figures 3D,F)—can be traced back to a generalization effect increasing at the increase of phobic symptoms (as expected from phobic patients; American Psychiatric Association, 2013) along a continuum.

The preferences expressed about the locomotion patterns (Figures 3G,H) are very different from those expressed for both hairiness and body/leg size. Indeed, in line with previous studies (Davey, 1991; Lindner et al., 2019), the vast majority of participants—regardless of their level of fear of spiders—considered spiders as dangerous when moving according to a spider-like pattern. In addition to this, the different perceptual preferences characterizing spiders shown in a frontal orientation from those shown in a lateral or posterior orientation (Supplementary Figures S1, S2) support previous findings showing that spiders crawling toward the participant elicit reactions different from the withdrawing ones (Lindner et al., 2019). In this regard, the feature most heavily affected by a spider’s orientation was dangerous hairiness, whereas preferences concerning the body/leg size and the locomotion almost overlapped (Supplementary Figures S1, S2).

Finally, the possibility of modeling flying spiders—which should have always been considered harmless to coherently mirror dangerous ones— has confused participants, resulting in the highest inter-individual incoherence among the three features (Figure 3I). This inter-individual incoherency in preferences could be explained by the locomotion pattern which is the only perceptual feature that could break realism since it ranged from a spider-like pattern to an impossible butterfly-like pattern.

As a final consideration, while these results are supported by the strengths coming from the introduction of an objective behavioral assessment based on augmented reality (more objective than the self-report tools used so far in the scientific literature about phobic perceptual preferences), the whole study inevitably comes with some noteworthy limitations that impose caution in generalizing its results. These limitations—better detailed in the next section—will be overcome only by a wider replication of the study, made possible by the full sharing of its complete data.

### Limitations

The main limitation of the present experiment is its high aversiveness for the most severely spider-fearful people, which resulted in a sample lacking the most phobic participants (SPQ score > 25). In fact, the experimental setup—consisting of tridimensional, realistic spiders floating in the dark—was perceived to be too stressful for the most spider-fearful volunteers, one of whom left the experiment before its end. Even if participants were informed and reassured about the virtuality of phobic stimuli, they were nevertheless frightened by its realism: this irrationality is coherent with the clinical definition of specific phobia (American Psychiatric Association, 2013).

This inevitable limitation, while confirming the ecological validity of the experimental setting (which is nevertheless different from a real-life scenario), imposes caution in generalizing the results to a wider population before further replications of the study. In addition to that, the higher objectiveness of behavioral assessments with respect to self-report questionnaires comes at the cost of a potential failure to capture the full complexity of arachnophobia symptoms, which are thought to emerge from a mixture of cognitive, behavioral, and physiological factors (Frumento et al., 2021, 2022).

Another limitation of this study is the unbalanced distribution of men and women among participants included in the final sample, being, 12 and 23, respectively. Even if this distribution is in line with the higher prevalence of specific phobias among women (Eaton et al., 2018), it could still fail to assess gender-related biases. Finally, stimuli were administered in a fixed sequence; even if this sequence was previously pseudo-randomized to minimize possible biases, it could still represent a potential confounding factor that impacts the study’s internal validity.

## Conclusion

The present study is the first one attempting to objectively assess the preferences in perceptual features that make a prototypical phobic stimulus (a spider) more or less fearful and to link these preferences to arachnophobia symptoms. This topic has been so far investigated mostly through subjective self-reports, which are not as related as commonly thought to behavioral and physiological parameters (Baldini et al., 2022; Frumento et al., 2022) and could fail to address complex relationships between variables. With respect to the previous scientific literature, the use of augmented reality made it possible to change the perceptual features of spiders in real time, thus reaching the best compromise between ecological validity and experimental manipulation (Iannizzotto et al., 2024). In fact, the holographic tridimensional spiders were highly realistic, as confirmed by the aversiveness of the experiment reported by the most spider-fearful participants (one of whom felt the need to abandon the experimental session after the first trial).

On average, spiders modified to be dangerous were hairy, thick, and moving following spider-like locomotion (Figure 3L), whereas spiders modified to be harmless showed opposite features (Figure 3M). However, these preferences underlie more complex relationships between each perceptual feature and the level of fear of spiders. In particular, inter-individual incoherency in hairiness preferences was increasing with the increase of fear of spiders, consistent with the generalization tendency described in DSM 5 (American Psychiatric Association, 2013). In addition, the locomotion of dangerous spiders showed a ceiling effect consistent with previous evidence showing that spider-like movements are the most feared, regardless of phobia level (Lindner et al., 2019). This suggests that the generalization effect is only valid for realistic features and thus does not apply to spiders with a butterfly-like locomotion pattern.

Considering that flying spiders do not exist in nature, this last result assumes even more relevance. In fact, a rationality-driven decision process would consider a flying spider to be more dangerous than a non-flying one—the more the abilities, the more the potential harm. Nevertheless, holographic spiders induced a higher fear when they were mostly resembling the unique perceptual features of a prototypical spider that is clearly distinguishable from all other insects for its hairiness, movement pattern, and thick body/leg proportions.

These results suggest that conscious cognitive processes and rational thinking are not driving the preferences concerning which perceptual features make a spider dangerous or harmless. In fact, both spider-fearful and non-spider-fearful participants considered spiders dangerous when their appearance matched that of an ancestral menace (e.g., crawling in a spider-like pattern). In addition to that, the group of spider-fearful participants felt threatened by a wider range of spidery features (e.g., low hairiness or slim body/leg size) that do not seem to rely on any rational ground (e.g., none of these features is a reliable marker of spider poisonousness or aggressiveness).

These unconsciously driven preferences support the idea that spider phobia stems from innate mechanisms aimed at quick and dirty detection of evolutionary-relevant animals (LeDoux, 1994). However, the present study is not designed to unambiguously discriminate whether these preferences are culturally or biologically driven. Future studies implementing a subliminal administration of spider-like stimuli differing for perceptual features could provide further information about the nature of these preferences by getting around to conscious processing—most likely affected by cultural biases.

Finally, the recent frontier of generative artificial intelligence opens up the possibility of using the pictures produced for each trial (made publicly available together with the corresponding raw data) to train systems aimed at creating phobic stimuli that elicit different intensities of fear to improve the acceptability of exposure therapies and the generalizability of their therapeutic outcomes.

## Data availability statement

The datasets presented in this study can be found in online repositories. The names of the repository/repositories and accession number(s) can be found below: The Unity software for the “spider-modifier” used in the experiment is publicly available on GitHub repository (https://github.com/M45K/RitualOfChud). Data and analysis scripts (performed in R software) are publicly available at the Open Science Framework repository (https://osf.io/73s2q/?view_only=c9b217b1ad3b433faa271a496cc08f71).

## Ethics statement

The studies involving humans were approved by Comitato Bioetico dell’Università di Pisa. The studies were conducted in accordance with the local legislation and institutional requirements. The participants provided their written informed consent to participate in this study.

## Author contributions

SF: Conceptualization, Data curation, Formal analysis, Writing – original draft. PF: Data curation, Formal analysis, Methodology, Writing – review & editing. ML: Supervision, Writing – review & editing. DM: Supervision, Writing – review & editing, Data curation, Methodology. AG: Conceptualization, Funding acquisition, Project administration, Resources, Supervision, Writing – review & editing.

## Footnotes

^1^ https://www.cyberfreak.it/

^2^ https://osf.io/73s2q/?view_only=c9b217b1ad3b433faa271a496cc08f71

^3^ https://github.com/M45K/RitualOfChud
