# Supplementary material for: The fear of spiders: perceptual features assessed in augmented reality
Source: Front Behav Neurosci. 2024 Feb 21;18:1355879. doi: 10.3389/fnbeh.2024.1355879 (PMC10915047; doi:10.3389/fnbeh.2024.1355879)

# Supplementary materials

**Supplementary Figure 1**: perceptual features that make spiders dangerous, distinguished for spider’s orientation


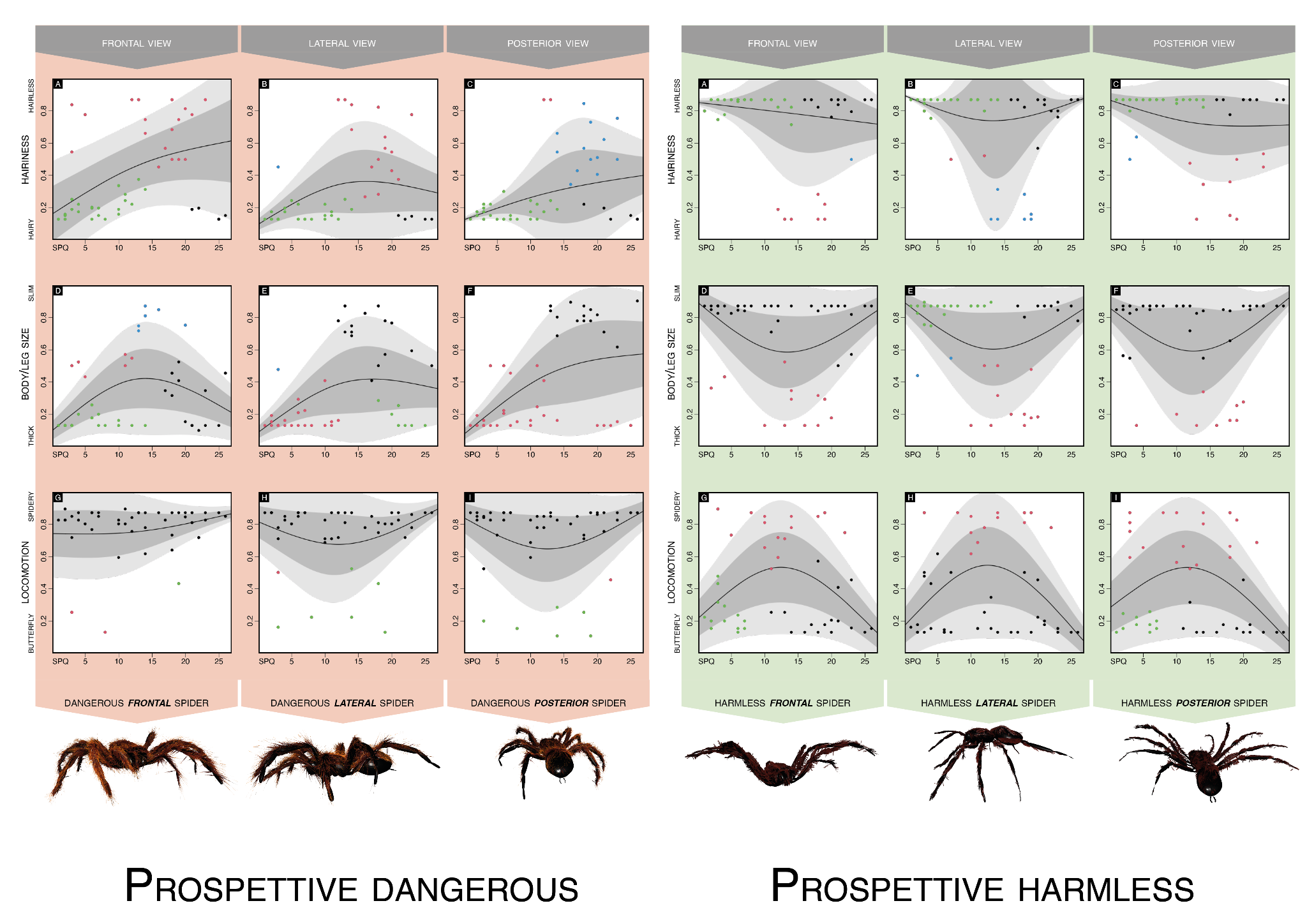


**Supplementary Figure 2**: perceptual features that make spiders harmless, distinguished for spider’s orientation


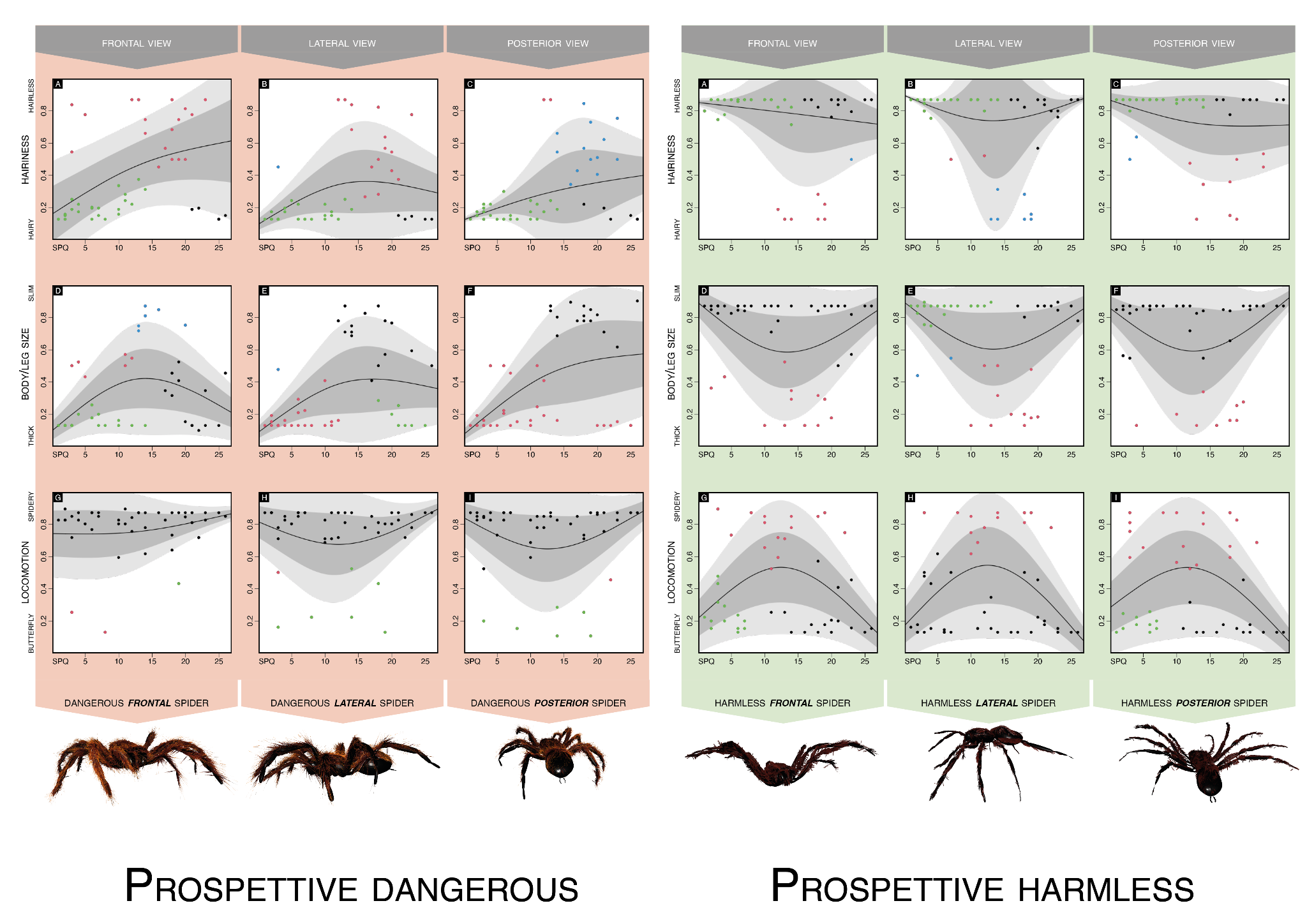


**Supplementary Figure 3**: replication of main Figure 2 based on means


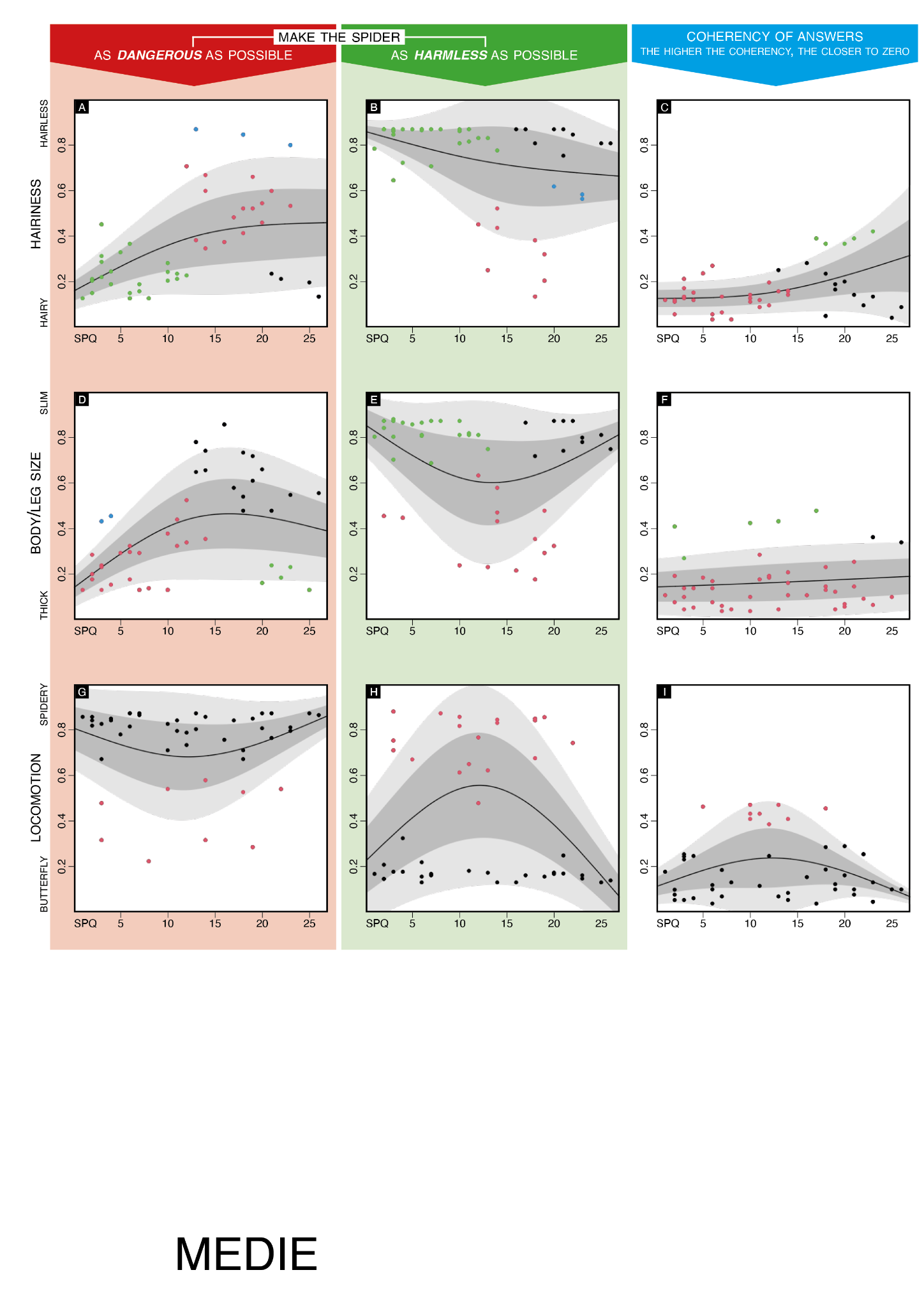


**Supplementary Figure 4**: replication of main Figure 2 based on medians


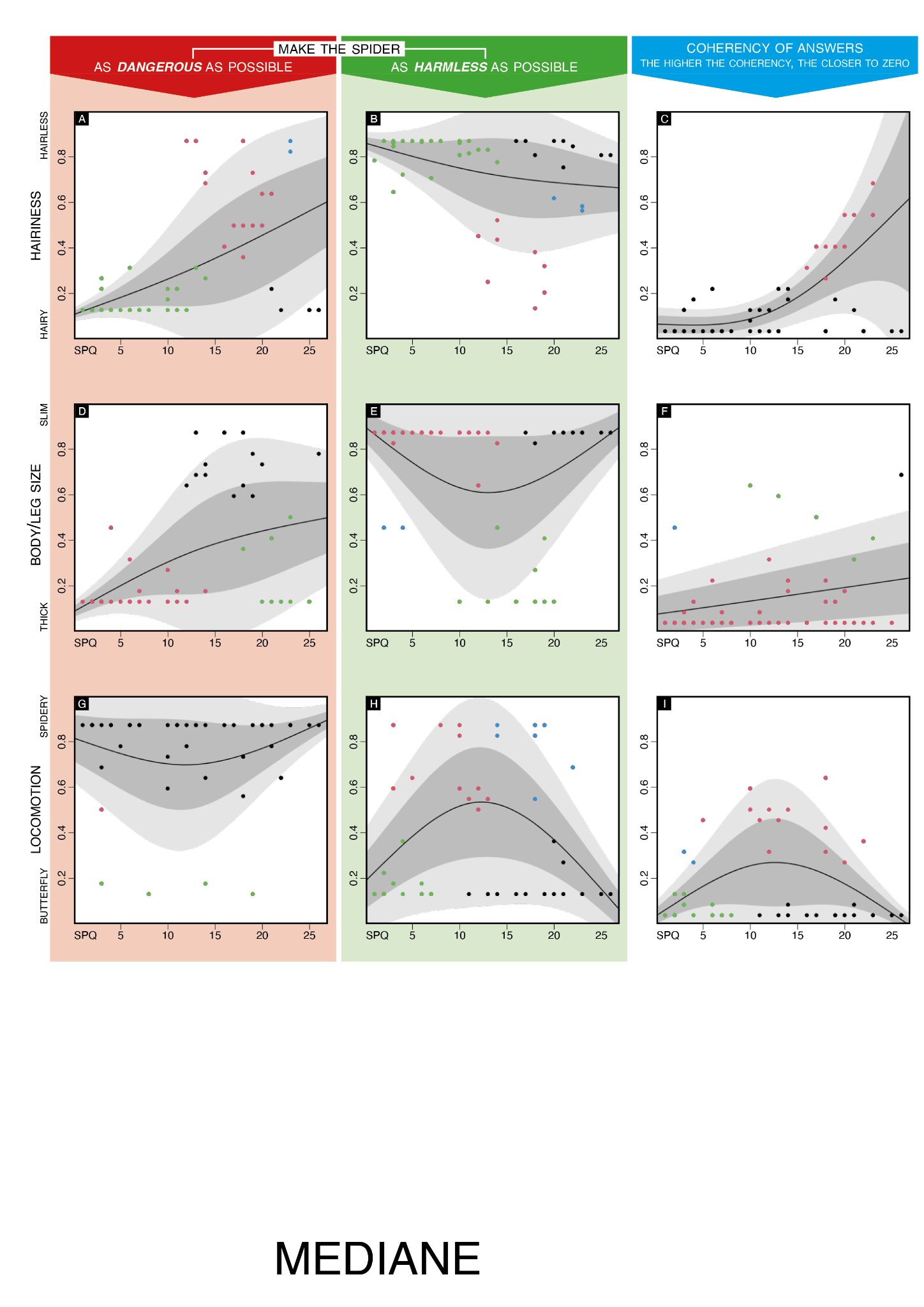


**Supplementary Figure 5**: replication of main Figure 2 with clustering based on all features simultaneously (including SPQ)


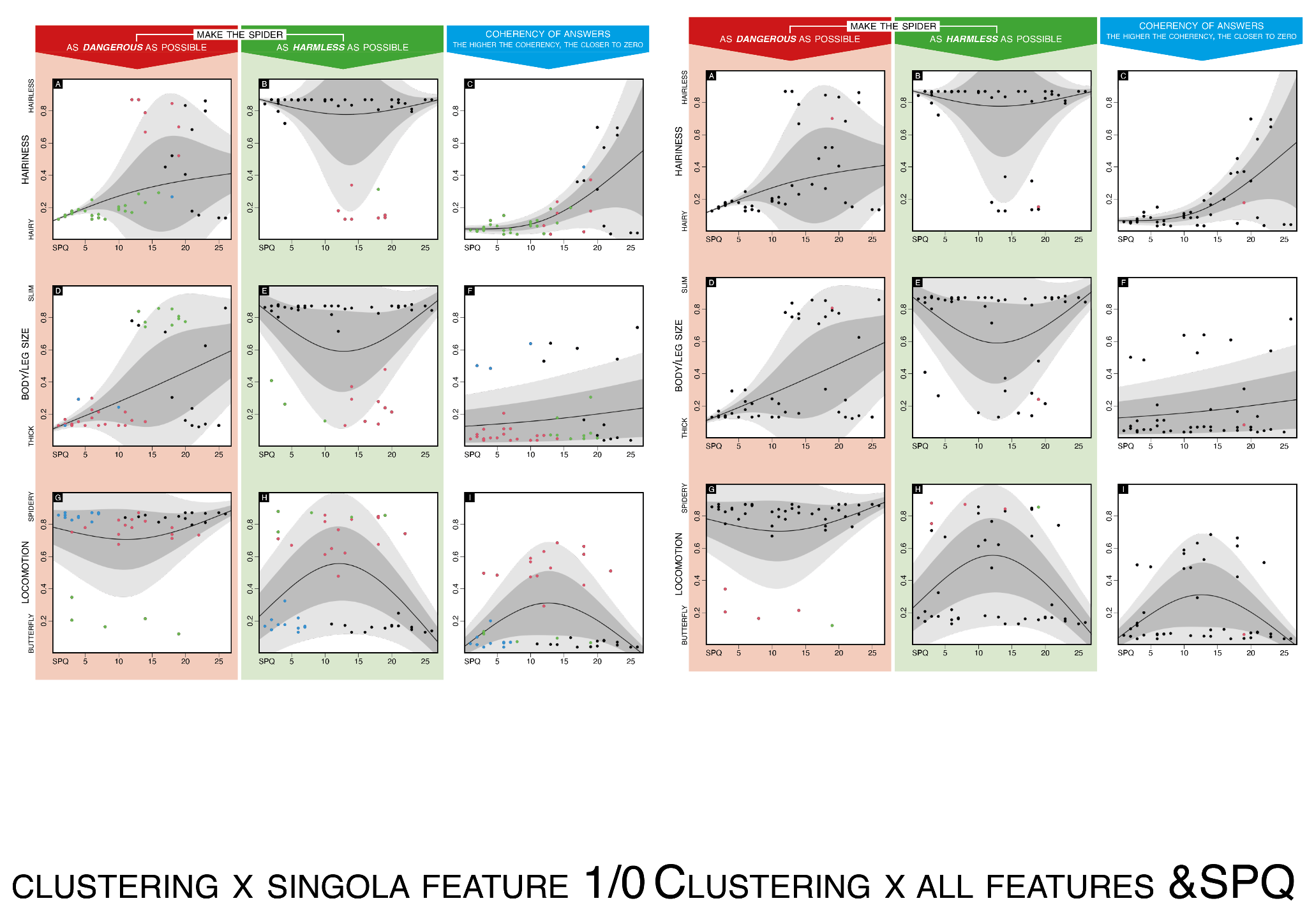


**Supplementary Figure 6**: replication of main Figure 2 with clustering based on all perceptual features simultaneously (excluding SPQ)


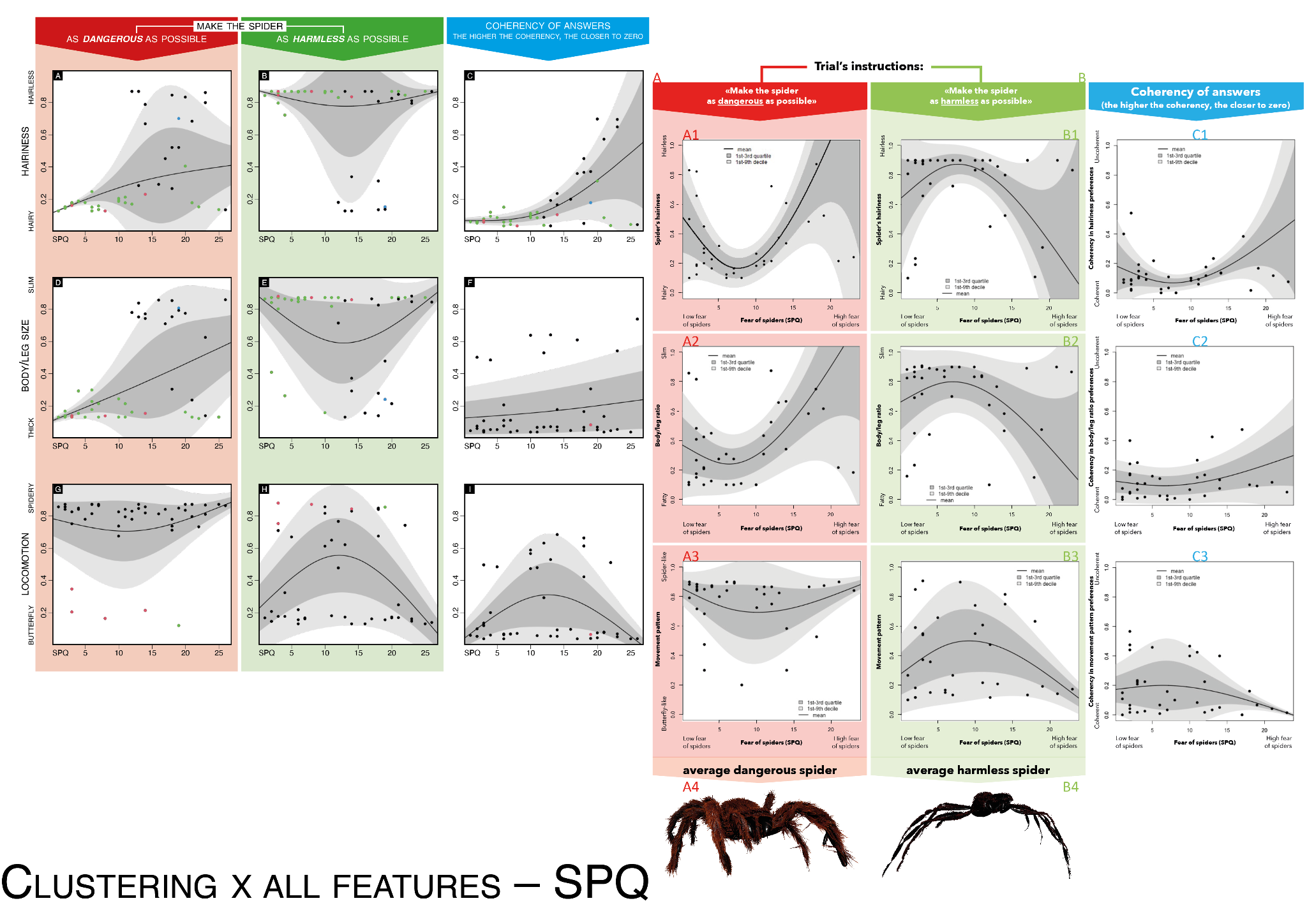


**Supplementary Figure 7**: replication of main Figure 2 with clustering based on each perceptual feature in both its harmless and dangerous version (excluding SPQ)


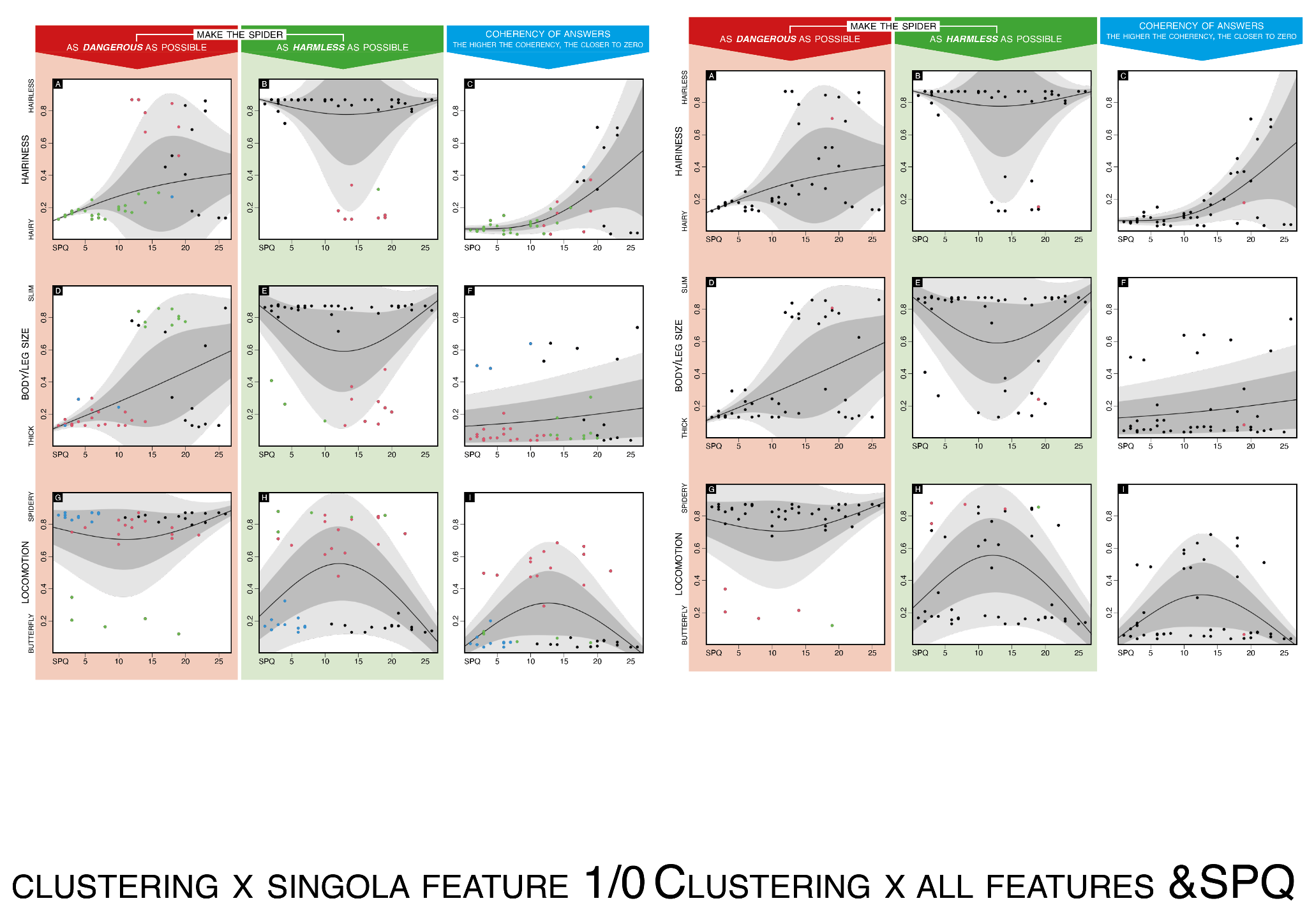

Supplement: Supplementary file 1 [file Data_Sheet_1.docx]
