# Supplementary material for: The fear of spiders: perceptual features assessed in augmented reality
Source: Front Behav Neurosci. 2024 Feb 21;18:1355879. doi: 10.3389/fnbeh.2024.1355879 (PMC10915047; doi:10.3389/fnbeh.2024.1355879)

RATIONALE

Are **arachnophobic** persons afraid of all spiders, or they share *preferences* about their **perceptual features**?

SAMPLE

56 volunteers differing for **self-reported** levels of fear for spiders (SPQ)

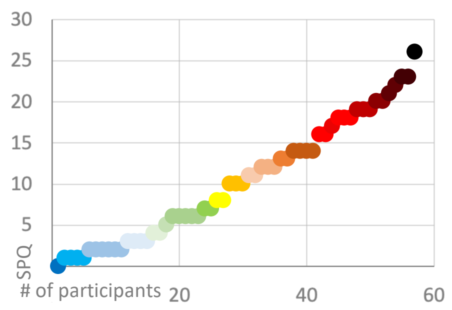

EXPERIMENTAL SETTING IN AUGMENTED REALITY

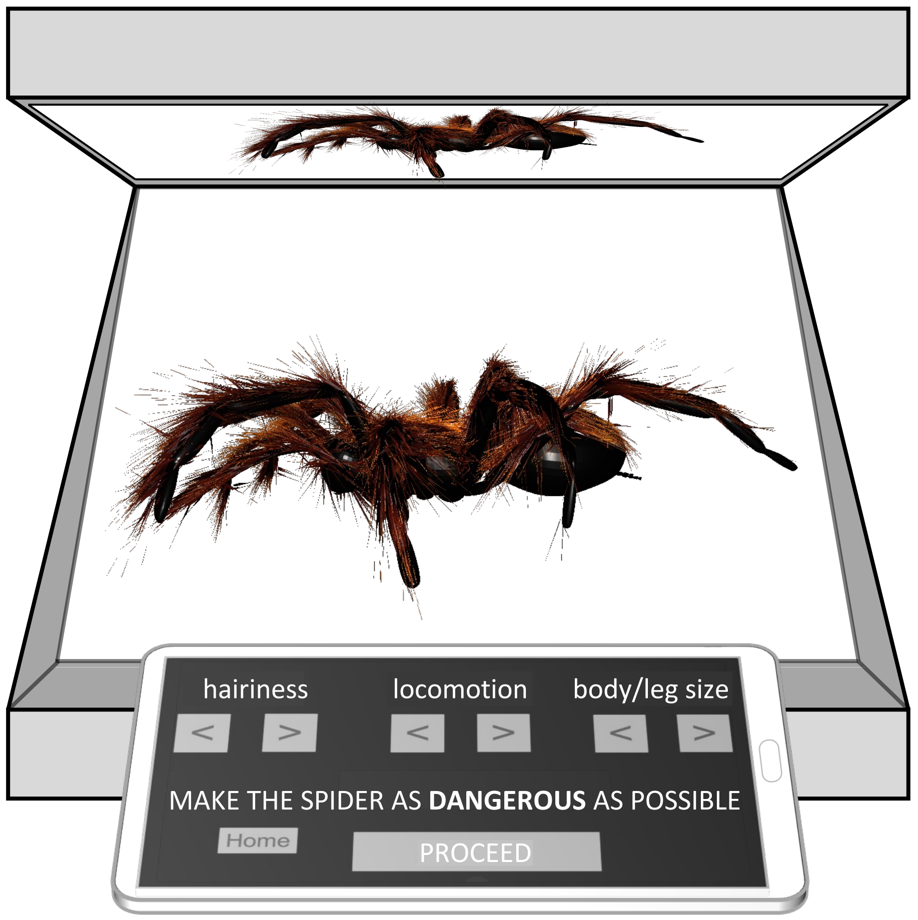

RESULTS

Arachnophobic persons tend to **generalize** fear. However, *on average*:

- **dangerous** spiders are *fat*, *hairy*, and *crawl* like a spider

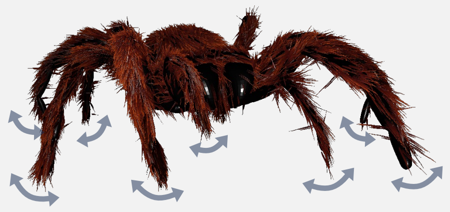

- **harmless** spiders are *thin*, *hairless*, and *fly* like a butterfly

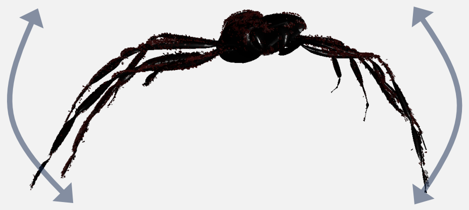

Supplement: Supplementary file 2 [file Image_1.pdf]
